# Modulation of Tetraspanin 32 (TSPAN32) Expression in T Cell-Mediated Immune Responses and in Multiple Sclerosis

**DOI:** 10.3390/ijms20184323

**Published:** 2019-09-04

**Authors:** Salvo Danilo Lombardo, Emanuela Mazzon, Maria Sofia Basile, Giorgia Campo, Federica Corsico, Mario Presti, Placido Bramanti, Katia Mangano, Maria Cristina Petralia, Ferdinando Nicoletti, Paolo Fagone

**Affiliations:** 1 Department of Biomedical and Biotechnological Sciences, University of Catania, 95123 Catania, Italy; 2 IRCCS Centro Neurolesi Bonino Pulejo, C.da Casazza, 98124 Messina, Italy

**Keywords:** TSPAN32, tetraspanins, T cell responses, multiple sclerosis

## Abstract

Tetraspanins are a conserved family of proteins involved in a number of biological processes including, cell–cell interactions, fertility, cancer metastasis and immune responses. It has previously been shown that TSPAN32 knockout mice have normal hemopoiesis and B-cell responses, but hyperproliferative T cells. Here, we show that TSPAN32 is expressed at higher levels in the lymphoid lineage as compared to myeloid cells. In vitro activation of T helper cells via anti-CD3/CD28 is associated with a significant downregulation of TSPAN32. Interestingly, engagement of CD3 is sufficient to modulate TSPAN32 expression, and its effect is potentiated by costimulation with anti-CD28, but not anti-CTLA4, -ICOS nor -PD1. Accordingly, we measured the transcriptomic levels of TSPAN32 in polarized T cells under Th1 and Th2 conditions and TSPAN32 resulted significantly reduced as compared with unstimulated cells. On the other hand, in Treg cells, TSPAN32 underwent minor changes upon activation. The in vitro data were finally translated into the context of multiple sclerosis (MS). Encephalitogenic T cells from Myelin Oligodendrocyte Glycoprotein (MOG)-Induced Experimental Autoimmune Encephalomyelitis (EAE) mice showed significantly lower levels of TSPAN32 and increased levels of CD9, CD53, CD82 and CD151. Similarly, in vitro-activated circulating CD4 T cells from MS patients showed lower levels of TSPAN32 as compared with cells from healthy donors. Overall, these data suggest an immunoregulatory role for TSPAN32 in T helper immune response and may represent a target of future immunoregulatory therapies for T cell-mediated autoimmune diseases.

## 1. Introduction

Tetraspanins are evolutionarily conserved cell-membrane proteins involved in a variety of biological functions, from cell adhesion to intercellular communication and signaling. The tetraspanin family counts 33 different members in the human species: some of them are ubiquitously expressed, others are tissue specific [1].

The tetraspanin structure consists of four transmembrane (TM) domains, with TM domains 1 and 2 flanking a small extracellular loop (SEL), and TM 3 and 4 flanking a large extracellular loop (LEL). Typically, the extracellular regions are involved in protein–protein interactions with other membrane proteins and external ligands, such as integrins, while the intracellular domains mediates the activation of signaling pathways. Tetraspanins are clustered in membrane microdomains, known as tetraspanin-enriched microdomains, that regulate the intracellular transmission of external stimuli [2].

Tetraspanins significantly contribute to different processes of the immune system, as shown from their interactions with leukocyte proteins such as integrins and immunoreceptors, which allows these proteins to modulate immune responses and processes such as antibody production, T cell proliferation, leukocyte migration and extravasation. Some members of the superfamily (e.g., CD9, CD37 and CD81) have been extensively studied for their immune-modulatory role in the co-stimulation and T cell polarization processes [2].

In light of their role in regulating immune responses, the tetraspanin superfamily can be a potential target for novel therapeutic approaches. Recent studies have investigated the contribution of some members of the tetraspanin family to autoimmune diseases, in particular to multiple sclerosis (MS), showing a possible involvement in its pathogenesis by controlling the transmigration of lymphocytes and monocytes to the central nervous system (CNS) [3,4]. However, little is known about the pathophysiological role of TSPAN32 (a.k.a. Tssc6) in the regulation of immune responses.

In a previous study, Tssc6gt/gt T cells bearing a null mutation of the Tssc6 allele showed enhanced responses to stimulation due to increased IL-2 production, suggesting that Tssc6 may play a role in the negative regulation of peripheral T-lymphocyte proliferation [5].

The present study focuses on the characterization of TSPAN32 in T cell responses and provides the first proof of concept for a possible role of TSPAN32 in the immune dysregulation observed in MS, defining this molecule as a potential translational target for further studies.

## 2. Results

### 2.1. TSPAN32 Expression Analysis in Immune Cells

**Figure 1:**
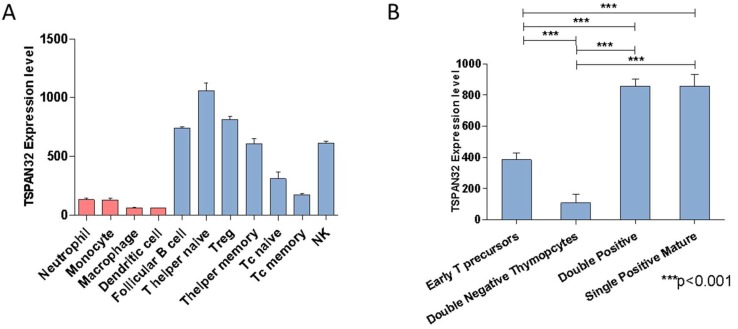
Expression of TSPAN32 in murine immune cells. (**A**) Expression of TSPAN32 in murine immune lineages was evaluated by interrogating the GSE15907 microarray dataset. (**B**) Modulation of the transcriptomic levels of TSPAN32 during T cell development, as determined from the GSE15907 dataset. Data are shown as normalized mean ± SD and statistical analysis performed using one-way ANOVA followed by Bonferroni multiple test correction.

Analysis of TSPAN32 expression levels in leukocyte populations revealed that TSPAN32 is expressed at higher levels in the lymphoid lineage as compared with myeloid cells. Among the lymphoid cells, cytotoxic T cells expressed lower levels of TSPAN32 while naïve T helper cells had the highest expression (Figure 1A). During T cell development, TSPAN32 expression was acquired in the later phases of thymocyte development (i.e., at the stage of double positive cells). In particular, double negative thymocytes showed the lowest expression, while both double positives and single positive mature thymocytes showed the highest expression levels (Figure 1B).

### 2.2. Tetraspanins Expression in T Cell Activation

**Figure 2:**
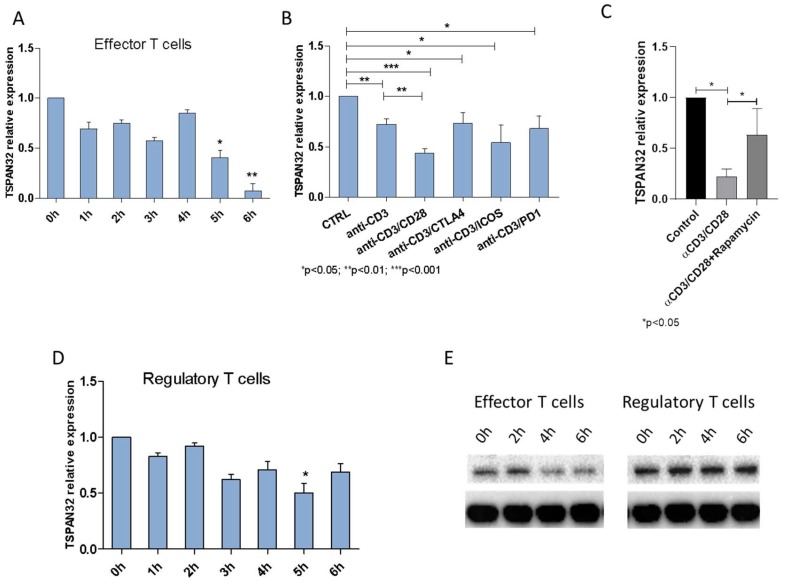
Expression of TSPAN32 during human T cell activation. (**A**) Expression of TSPAN32 was evaluated via real-time PCR, in CD4+ T effector cells stimulated at different time points with anti-CD3/CD28 antibodies or (**B**) with anti-CD3 alone or in combination with anti-CD28, anti-CTLA4, anti-PD1 and anti-ICOS antibodies for 36 h (*n* = 3 independent replicates). (**C**) TSPAN32 levels in CD4+ effector T cells upon anti-CD3/CD28 stimulation, in the presence or absence of rapamycin 200 nM, were evaluated by real-time PCR (*n* = 3 independent replicates). (**D**) TSPAN32 expression levels were evaluated at different time points upon activation of Treg cells via real-time PCR (*n* = 3 independent replicates). (**E**) TSPAN32 protein levels were determined by western blot upon activation of effector and regulatory T cells at different time points (pooled proteins of cells from 3 healthy donors). Data are shown as normalized mean ± SD and statistical analysis performed using one-way ANOVA followed by Bonferroni multiple test correction.

Analysis of TSPAN32 expression levels during T cell activation showed a time-dependent decrease in the levels of TSPAN32 in effector T cells following anti-CD3/CD28 stimulation. In particular, a significant reduction in TSPAN32 levels was observed starting at 5 h post stimulation (Figure 2A).

Anti-CD3 stimulation was sufficient to significantly downregulate TSPAN32 (*p* < 0.05 vs. the control unstimulated cells), and its effect was potentiated by co-stimulation with anti-CD28 (*p* < 0.001 vs. the control unstimulated cells and *p* < 0.01 vs. the anti-CD3 stimulated cells) (Figure 2B). No significant differences were observed in TSPAN32 levels after anti-CD3 stimulation and co-stimulation with anti-CTLA4, anti-ICOS or anti-PD1 antibodies (Figure 2B).

Since CD28-mediated signaling depends on the PI3K/Akt/mTOR pathway, we wanted to verify whether mTOR could be involved in the modulation of TSPAN32 expression. As expected, treatment of T cells with the mTOR inhibitor rapamycin were shown to significantly increase the levels of TSPAN32 (*p* < 0.05) (Figure 2C).

In the Treg subset of CD4+ lymphocytes, a moderate decrease in TSPAN32 expression levels was also observed upon activation, which reached statistical significance only at 5 h post stimulation (*p* < 0.05 vs. the control unstimulated cells) (Figure 2D). Significantly lower levels of TSPAN32 were observed in T effector cells as compared with Treg cells at 5 and 6 h post stimulation (*p* < 0.05).

The modulation of TSPAN32 in both effector and regulatory cells was further confirmed at the protein level. As shown in Figure 2E, a marked reduction of TSPAN32 could be observed in effector T cells at 4 h post activation, while no modulation was observed in Treg cells.

### 2.3. Tetraspanins Expression in T Cell Polarization

**Figure 3:**
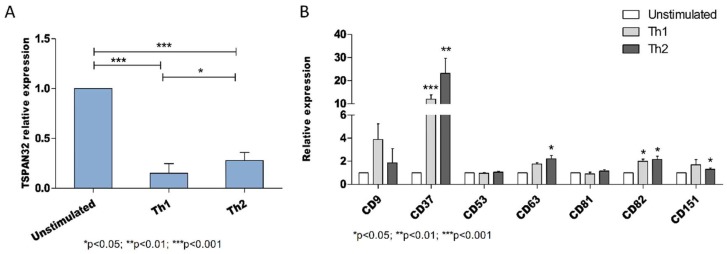
Expression of tetraspanins in human T cell polarization. (**A**) Expression of TSPAN32 was evaluated by real-time PCR in polarized Th1 and Th2 cells and unstimulated cells (*n* = 3 independent replicates). (**B**) Expression of CD9, CD37, CD53, CD63, CD81, CD82 and CD151 in polarized Th1 and Th2 cells and unstimulated cells as evaluated by real-time PCR (*n* = 3 independent replicates). Data are shown as normalized mean ± SD and statistical analysis performed using one-way ANOVA followed by Bonferroni multiple test correction.

Next, we determined the transcriptomic levels of TSPAN32 in polarized T cells under Th1 and Th2 conditions. TSPAN32 in both Th1 and Th2 cell subsets was significantly reduced in comparison with unstimulated cells (*p* < 0.001 for both Th1 and Th2 cells as compared with the control unstimulated cells) (Figure 3A). Moreover, a significantly lower TSPAN32 expression was observed in Th1 cells (*p* < 0.05) as compared with Th2 cells.

The expression levels of other members of the tetraspanin family were also evaluated for comparison and CD9, CD37, CD53, CD63, CD81, CD82 and CD151 were considered. Among them, a significant increase could be observed in both Th1 and Th2 cells for CD37 and CD82, while a significant increase in Th2 cells as compared with unstimulated cells was observed for CD63 and CD151 (Figure 3B). The expression of CD9, CD53 and CD81 did not show significant variation between the unstimulated and the polarized cells (Figure 3B).

**Figure 4:**
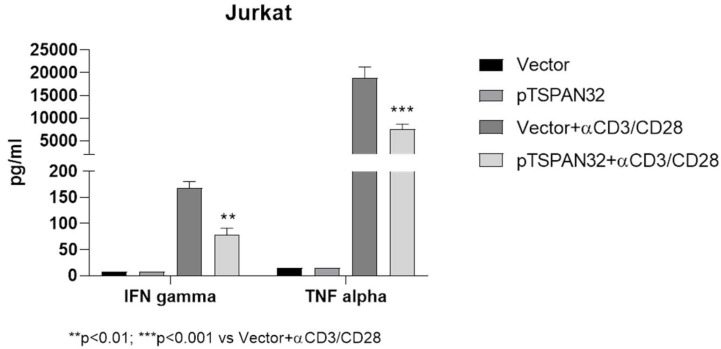
Effect of TSPAN32 overexpression in Jurkat cells. Following transient transfection of Jurkat cells with a DNA plasmid encoding for TSPAN32 (pTSPAN32) or the empty plasmid, cells were stimulated with anti-CD3/CD28 for 24 h and the concentrations of TNF-alpha and IFN-gamma in the supernatant were determined by ELISA. Data are shown as normalized mean ± SD and statistical analysis performed using one-way ANOVA followed by Bonferroni multiple test correction.

In Jurkat T cells, overexpression of TSPAN32 (induced by transient transfection with a TSPAN32-encoding plasmid) was associated with a significant reduction in the production of the pro-inflammatory cytokines TNF-alpha and IFN-gamma upon cell activation (Figure 4).

### 2.4. TSPAN32 in Multiple Sclerosis

**Figure 5:**
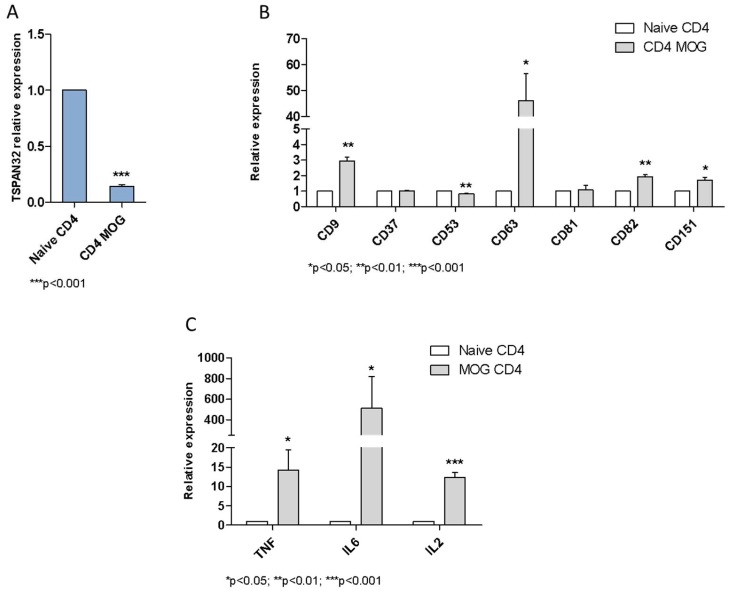
Modulation of TSPAN32 in experimental allergic encephalomyelitis (EAE). (**A**) Expression of TSPAN32 was evaluated using real-time PCR in CD4+ T cells isolated from the brains and spinal cords of mice with EAE. Control cells are represented by CD4+ T cells isolated from the spleens of sham mice (*n* = 3 replicates, each of 3 pooled animals). (**B**) Expression of CD9, CD37, CD53, CD63, CD81, CD82 and CD151 was evaluated using real-time PCR in CD4+ T cells isolated from the brains and spinal cords of mice with EAE. Control cells are represented by CD4+ T cells isolated from the spleens of sham mice (*n* = 3 replicates, each of 3 pooled animals). (**C**) Expression of TNF-alpha, IL-6 and IL-2 was evaluated by real-time PCR in CD4+ T cells isolated from the brains and spinal cords of mice with EAE. Control cells are represented by CD4+ T cells isolated from the spleens of sham mice (*n* = 3 replicates, each of 3 pooled animals). Data are shown as normalized mean ± SD and statistical analysis performed using one-way ANOVA followed by Bonferroni multiple test correction.

The expression of TSPAN32 was assessed in CD4+ T cells from a model of experimental autoimmune encephalomyelitis (EAE). Encephalitogenic T cells from MOG-induced EAE mice showed significantly lower levels of TSPAN32 (*p* < 0.001) (Figure 5A) and increased levels of CD9 (*p* < 0.01), CD63 (*p* < 0.05), CD82 (*p* < 0.01) and CD151 (*p* < 0.05) (Figure 5B). A decrease in TSPAN32 levels was also observed for CD53 in T cells from MOG-immunized mice (*p* < 0.01) (Figure 5B). The analysis of the expression levels of pro-inflammatory cytokines in CD4 T cells from MOG-immunized mice showed significantly higher levels for TNFα (*p* < 0.05), IL-6 (*p* < 0.05) and IL-2 (*p* < 0.001) (Figure 5C).

**Figure 6:**
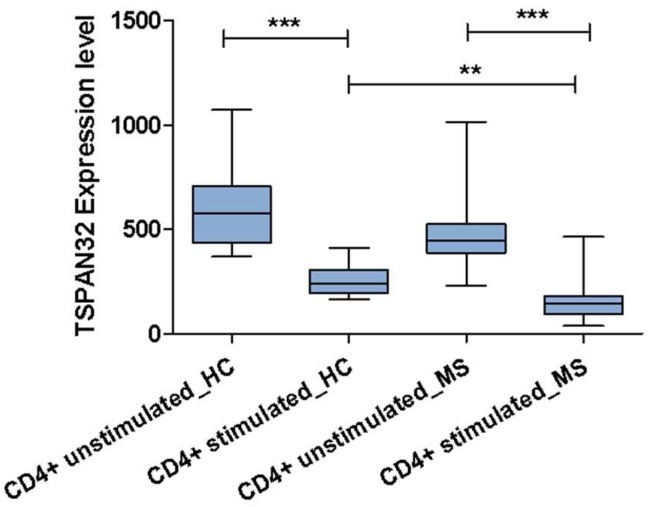
Modulation of TSPAN32 in multiple sclerosis. Expression of TSPAN32 resting and activated CD4+ T cells from 14 relapsing-remitting MS patients and 14 healthy donors, as obtained from the GSE78244 dataset. Data are shown as normalized mean ± SD and statistical analysis performed using one-way ANOVA followed by Bonferroni multiple test correction. ** *p* < 0.01, *** *p* < 0.001.

Finally, TSPAN32 expression was analyzed in lymphocytes from healthy controls (HC) and MS patients before and after anti-CD3/CD28 stimulation. In both the HC and MS, stimulation reduced TSPAN32 expression levels (*p* < 0.001). Moreover, the stimulated lymphocytes from MS patients showed lower TSPAN32 levels than the stimulated lymphocytes from HC (*p* < 0.01) (Figure 6). 

## 3. Discussion

Tetraspanins are involved in the regulation of different steps of the immune response, such as balancing the activation threshold of immune receptors, modifying surface expression and the spatial arrangement of adhesion molecules and their subsequent intracellular signaling to control APCs (Antigen-Presenting Cells migration. Furthermore, tetraspanins participate in the process of antigen processing and have an important role in priming naïve T cells through the control of T cell co-stimulation and MHC-II-dependent antigen presentation [6].

Several members of the family have been shown to play a crucial role in the adaptive immune system. For instance, CD81 plays a key role in the formation of the immune synapse as it mediates the contact between the APC and the T cells [7,8], while CD37 and CD151 are implicated in antigen presentation and costimulatory inputs [9].

In a Tssc6 (TSPAN32)-deficient mouse model, T cell activation was altered, while leucocyte development was normal. Thus, TSPAN32 seems to not be required for the development of the hematopoietic system, but it may play a role in the negative regulation of peripheral T-lymphocyte activation [5]. In mice knockout for both CD37 and TSPAN32, in vitro T cell proliferative responses and dendritic cell stimulation capacity were significantly augmented compared with single knockout, eliciting a cooperative role for these two proteins in cellular-mediated immunity [10]. These findings support the hypothesis of an immune-regulatory role for TSPAN32.

In the present work, we have characterized the modulation of TSPAN32 in T cell-mediated immune responses and in multiple sclerosis by means of in silico, in vitro and ex vivo analysis. The use of whole-genome expression data has been largely used by us and other groups [11,12,13,14] to identify pathogenic pathway and novel therapeutic targets in a variety of clinical settings, including autoimmune diseases [15,16,17,18,19], cancer [20,21,22,23,24,25,26,27], fibrotic diseases [28] and neurological and infectious diseases [29,30].

We show here for the first time that TSPAN32 is prevalently expressed in lymphoid lineage, with the highest expression in naïve T-helper cells. In particular, we observed that TSPAN32 expression is acquired in the later phases of the T maturation process (double positive and single positive mature).

To assess the relationship between TSPAN32 expression and T cell activation, different experimental conditions were tested. In vitro activation of T-helper cells via anti-CD3 was associated with a significant downregulation of TSPAN32, and its effect was potentiated by costimulation with anti-CD28, but not anti-CTLA4, anti-ICOS or anti-PD1. Since CD28 signaling is dependent on the PI3K/Akt/mTOR pathway, we investigated whether mTOR could promote a modulation in TSPAN32 levels. In accordance with our hypothesis, treatment of T cells with rapamycin was associated with significantly higher levels of TSPAN32. The PI3K/Akt/mTOR pathway physiologically plays a critical role in driving T cell differentiation and function. Indeed, upon rapamycin treatment, TCR engagement leads to T cell tolerance even in the presence of costimulation [31]. We have previously demonstrated the involvement of mTOR in the generation and progression of MS [17,32]. Administration of rapamycin to experimental allergic encephalomyelitis (EAE) Dark Agouti rats, a model of relapsing-remitting multiple sclerosis (RRMS), improved the clinical course of the disease, increasing the percentage of Tregs and reducing the number of CD8+ T cells. Along the same lines, administration of rapamycin to RRMS patients determined a significant reduction in plaque area size and improved Expanded Disability Status Scale (EDSS) score [33]. Based on the data presented here, it can be speculated that part of the effects of rapamycin in EAE/MS may be imputable to the modulation of TSPAN32 expression.

The measurement of the transcriptomic levels of TSPAN32 in polarized T cells showed that TSPAN32 expression was significantly reduced in polarized cells as compared to unstimulated cells. Interestingly, Th2 T cells showed a higher TSPAN32 expression than the Th1 subset. This may be explained by the anti-inflammatory role of Th2 T cells, as it has also been observed in MS and in experimental autoimmune encephalitis (EAE) [34]. Notably, HOXA3, a transcription factor promoting the expression of TSPAN32 [35], is also responsible for the M2 polarization in macrophages. This may suggest that the possible tolerogenic role of TSPAN32 may not be limited to lymphocytes [36].

It is worth pointing out that the analysis of the expression of other tetraspanins upon polarization showed a significant increase in CD37, CD63, CD82 and CD151 levels in Th1 and Th2 cells as compared with the unstimulated cells in accordance with their role in the T cell activation processes. We may speculate that TSPAN32 works as a brake for other costimulatory factors, including members of the tetraspanin family, thus regulating immune tolerance. Indeed, prediction analysis for proteins interacting with TSPAN32 identified CD63 and TSPAN7 as the top protein–protein partners for TSPAN32 (http://dcv.uhnres.utoronto.ca/FPCLASS/ppis/).

Finally, we have investigated the role of TSPAN32 in multiple sclerosis. Encephalitogenic T cells from MOG-induced experimental autoimmune encephalomyelitis (EAE) mice showed significantly lower levels of TSPAN32, which can be explained by a higher activity of T lymphocytes; the increased levels of CD9, CD53, CD82 and CD151 are concordant with the in vitro data, suggesting their role in the activation of the immune response. This is confirmed by the higher levels of pro-inflammatory cytokines (TNF-alfa, IL-6, IL-2) observed in MOG CD4 T cells, showing an inverse correlation with TSPAN32 expression.

Lastly, the analysis of TSPAN32 in CD4 T cells from healthy donors and MS patients confirmed the results gathered from the in vitro and vivo analyses. Stimulated CD4 cells showed significantly lower levels of TSPAN32 than the unstimulated counterparts; in addition, comparison between the CD4 T from healthy controls and MS patients showed that, upon stimulation, CD4 T cells from MS patients showed significantly lower transcriptional levels of TSPAN32.

As previously indicated, Tarrant et al. [5] have already demonstrated that CD4+ T cells from TSPAN32 knockout mice are hyper-proliferative and produce higher levels of IL2. Based on our data, we propose that T cells express a baseline level of TSPAN32 that hinders their proliferation and activation, promoting the maintenance of an inactive state. In the presence of antigens, the lymphocytes are stimulated and activated, resulting in a reduction of TSPAN32 levels via a possible activation of the mTOR pathway and an increase in CD9, CD53, CD82 and CD151, which are necessary for T-lymphocyte activation.

The disruption of the control switch provided by TSPAN32 may be one of the possible promoters of the inflammatory damage to myelin, offering not only an important insight into the pathogenesis of MS, but also novel therapeutic targets that may underlie potential primary drivers of autoimmunity.

To date, no drugs modulating the function of tetraspanins have been approved for the clinical use. However, different therapeutic approaches have been investigated in a variety of settings, including cancer [37] and microbial infections [38]. Tetraspanins can be targeted via multiple strategies, for instance using mAbs, recombinant soluble large extracelullar loops (sLELs) or RNA interference (RNAi) (reviewed by [39]). Hence, several opportunities for pharmacological intervention are available and could be exploited for the treatment of human diseases in the future.

## 4. Materials and Methods

### 4.1. TSPAN32 Expression Analysis in Murine Immune Cells

Analysis of TSPAN32 expression in different leucocyte subpopulations was performed by interrogating the GSE15907 microarray dataset. GSE15907 was generated as part of the Immunological Genome Project (ImmGen) [40]. Briefly, primary cells from multiple immune lineages were isolated ex vivo from young adult C57/B6 male mice (*n* = 3) and double-sorted to yield >99% purity. RNA was extracted and whole-genome transcriptomic levels were obtained using the Affymetrix 1.0 ST MuGene array platform (Santa Clara, California, U.S.).

### 4.2. Tetraspanins Expression in T Cell Activation

#### 4.2.1. Purification and Cultivation of Human CD4+ T Cells

Mononuclear cells were obtained from the peripheral blood of healthy donors (*n* = 3) by step-gradient centrifugation using Ficoll-Hypaque medium (Sigma Aldrich, Milano, Italy), as previously described [41]. CD4+CD25− T effector cells and CD4+CD25hi Treg were enriched by positive sorting using magnetic beads, obtaining a purity of >95%. Cells were stimulated with plate-bound anti-CD3/CD28 antibodies and 50 U/mL recombinant human IL2 (PeproTech, BDA S.r.l., Italy) for up to 6 h, and total RNA was collected every hour using TRIzol Reagent (Invitrogen, Milan, Italy). Unstimulated cells served as control cells.

In a separate set of experiments, effector T cells from three healthy donors were stimulated with anti-CD3 alone or in combination with anti-CD28, anti-CTLA4, anti-PD1 and anti-ICOS antibodies for 36 h. Unstimulated cells served as a negative control. At the end of the incubation period, total RNA was extracted and TSPAN32 expression was evaluated by real-time PCR. Two μg of total RNA was reverse-transcribed with a High-Capacity cDNA Reverse Transcription Kit (Applied Biosystems, Monza, Italy) in a 20 μL reaction solution, and real-time PCR was carried out using the SYBR Green PCR Master Mix (Applied Biosystems, Monza, Italy), 200 nM forward and 200 nM reverse primers and 20 ug cDNA. Gene expression was calculated using the formula: 2^−ΔΔ*C*t^, where ΔΔ*C*t = (*C*t_target gene_ − *C*t_beta-actin_) stimulated cells – (*C*t_target gene_ − *C*t_beta-actin_) control cells.

Proteins were extracted using M-PER lysis buffer (Thermo Fisher Scientific; Monza, Italy), following the manufacturer’s instructions. Protein concentrations were quantified using the Bio-Rad Protein Assay (Bio-Rad, Milan, Italy). Proteins were resolved by SDS-PAGE, followed by blotting to PVDF membranes (Immobilon-P transfer membrane; Millipore). PVDF membranes were then incubated in 5% bovine serum albumin (BSA) in phosphate buffered saline (PBS) for 1 h at room temperature. Afterwards, membranes were incubated with an anti-TSPAN32 primary antibody (1:2000; Thermo Fisher Scientific) overnight at 4 °C. Membranes were then incubated with HRP-conjugated anti-IgG secondary antibody (1:2000; Santa Cruz Biotechnology; Heidelberg, Germany) for 1 h at room temperature. In order to verify the equal loading of proteins, membranes were stripped and reprobed with HRP-conjugated glyceraldehyde 3-phosphate dehydrogenase (GAPDH) antibody (1:1000; Cell Signaling Technology, Milan, Italy). Images of protein bands were visualized using an ECL system (Luminata Western HRP Substrates; Millipore, Milan, Italy), acquired by ChemiDoc MP System (Bio-Rad) and quantified using ImageJ software (National Institutes of Health).

#### 4.2.2. Involvement of mTOR in TSPAN32 Expression

In order to determine the involvement of the mTOR pathway in the modulation of TSPAN32 expression following T cell activation, CD4+ T cells from three healthy donors were stimulated with plate-bound anti-CD3/CD28 antibodies (PeproTech, BDA S.r.l., Italy) for 4 h, alone or in the presence of 200 nM rapamycin. Total RNA was collected using TRIzol reagent (Invitrogen, Milan, Italy) for the subsequent determination of TSPAN32 expression by real-time PCR. Unstimulated cells served as control cells.

#### 4.2.3. Expression of TSPAN32 in Th1 and Th2 Cells

Expression levels of TSPAN32 and the tetraspanins, CD9, CD37, CD53, CD63, CD81, CD82 and CD151 were evaluated in Th1 and Th2 cells. CD45RA^high^ naïve CD4 T cells from three healthy donors were activated by plate-bound anti-CD3/CD28 and IL12 (1 ng/mL), IFN-gamma (10 ng/mL) and anti-IL4 (1 μg/mL), to differentiate Th1 cells, and anti-IL12 (5 μg/mL), anti-IFN-gamma (5 μg/mL) and IL4 (1 ng/mL) to differentiate Th2 cells for 48 h. At day 3, medium was replenished and cells were cultured for an additional 4 days. Afterward, cells were re-stimulated with anti-CD3/CD28 for 12 h and total RNA was isolated for subsequent real-time PCR.

#### 4.2.4. TSPAN32 Overexpression in Jurkat Cells

Jurkat T cells, Clone E6-1 (ATCC, TIB-152; obtained from American Type Culture Collection (ATCC)), were cultured in RPMI 1640 medium + 2 mM glutamine + 10% fetal calf serum (FCS) + penicillin (100 U/mL)/streptomycin (0.1 mg/mL) at a cell density of 0.5 × 106 cells/mL. Transient transfection of Jurkat cells with a DNA plasmid encoding for TSPAN32 (pTSPAN32) or the empty plasmid was performed using Lipofectamine (Life Technologies, Monza, Italy), following the manufacturer’s instructions. To generate the pTSPANB32 plasmid, the cDNA fragment containing the entire open reading frame of the TSPAN32 gene (GenBank accession number: NM_005705) was amplified from the human liver cDNA library (Invitrogen, Monza, Italy) by polymerase chain reaction (PCR), and the product was inserted into pcDNA3 vector (Invitrogen). At 72 h post transfection, cells were stimulated with anti-CD3/CD28 for 24 h, and supernatant collected for the determination of TNF-alpha and IFN-gamma by ELISA using commercially available kits (Invitrogen). Three independent experiments were performed.

### 4.3. TSPAN32 in Multiple Sclerosis

#### 4.3.1. Induction of EAE Induced by MOG in C57BL/6 Mice

Female 8- to 10-week-old C57BL/6 mice were purchased by ENVIGO RMS s.r.l. (San Pietro al Natisone, Udine, Italy) and kept under standard laboratory conditions with ad libitum access to food and water. The protection of animals used in the experiment complies with Directive 86/609/EEC, implemented by D.Lgs. 26/2014.

The animals were immunized by a subcutaneous injection of 200 μg of MOG35-55 (Genemed Synthesis Inc, San Francisco, CA, USA) emulsified in Complete Freund’s Adjuvant (CFA) with 1 mg of Mycobacterium tuberculosis H37RA (Difco, Detroit, MI, USA). The emulsion was administered in two sites, draining into the axillary lymph nodes. Pertussis toxin 200 ng/mouse (Calbiochem, Nottingham, UK) was injected intra-peritoneally (i.p.) on days 0 and 2 post immunization (Mangano et al., 2014). Mice were observed daily for clinical signs of EAE. At the peak of disease, mononuclear cells were isolated from brains and spinal cords using a 40–70% Percoll gradient, and CD4+ T cells were enriched by positive sorting using anti-CD4+ beads, with a final purity of >95%. Control CD4+ T cells were obtained from the spleens of sham immunized animals. Expression levels of the genes of interest were determined using real-time PCR. Primer sequences were designed in-house or obtained from the PrimerBank database (http://pga.mgh.harvard.edu/primerbank/).

Gene expression was calculated using the formula: 2^−ΔΔ*C*t^, where ΔΔ*C*t = (*C*t_target gene_ − *C*t_beta-actin_) encephalitogenic CD4 T cells – (*C*t_target gene_ − *C*t_beta-actin_) control cells.

#### 4.3.2. TSPAN32 Expression in CD4+ T Cells from MS Patients

Gene expression profiling of resting and activated CD4+ T cells from MS patients and healthy subjects was obtained from the GSE78244 dataset. TSPAN32 expression levels were evaluated in unstimulated cells and upon 24 h incubation with anti-CD3/CD28 antibodies. GSE78244 included data from 14 relapsing-remitting MS patients and 14 healthy donors. All patients were women who had undergone no immunomodulatory or immunosuppressive treatment in at least the 2 months before sampling, with the exception of one patient who received intravenous immunoglobulin 2 weeks prior to sampling. Purity of cells was >96% [42].

### 4.4. Statistical Analysis

Data are shown as normalized mean ± SD and statistical analysis was performed using either a Student’s *t*-test or one-way ANOVA followed by Bonferroni multiple test correction. GraphPad Prism software was used for the statistical analysis and generation of the graphs.

## 5. Conclusions

TSPAN32 is a member of the tetraspanin family involved in the regulation of cell-mediated immune responses. As compared to other tetraspanins such as CD9, CD63 and CD81 that promote antigen presentation and T cell signaling, TSPAN32 is oppositely modulated upon T cell activation. Our in silico, in vitro and ex vivo data suggest an immune-regulatory role for TSPAN32 and its possible involvement in the pathogenesis of MS. This study follows previous reports on TSPAN32 in immunity and represents a starting point for the ideation of possible new therapies for immunoinflammatory/autoimmune diseases, exploiting its immunoregulatory role in cellular immune responses.
